# In Vitro and In Vivo Evaluation of a Polycaprolactone (PCL)/Polylactic-Co-Glycolic Acid (PLGA) (80:20) Scaffold for Improved Treatment of Chondral (Cartilage) Injuries

**DOI:** 10.3390/polym15102324

**Published:** 2023-05-16

**Authors:** Arely M. González-González, Raymundo Cruz, Raúl Rosales-Ibáñez, Fernando Hernández-Sánchez, Hugo J. Carrillo-Escalante, Jesús Jiovanni Rodríguez-Martínez, Cristina Velasquillo, Daniel Talamás-Lara, Juan E. Ludert

**Affiliations:** 1 Department of Infectomics and Molecular Pathogenesis, Center for Research and Advanced Studies (CINVESTAV), Mexico City 07360, Mexico; arely.gonzalez@cinvestav.mx (A.M.G.-G.); jrcruz@cinvestav.mx (R.C.); daniel_talamas@hotmail.com (D.T.-L.); 2 Laboratorio de Ingeniería Tisular y Medicina Traslacional, Facultad de Estudios Superiores Iztacala, Universidad Nacional Autónoma de Mexico (UNAM), Mexico City 54090, Mexico; jesusjiovanni@outlook.com; 3 Unidad de Materiales, Centro de Investigación Científica de Yucatán, Mérida 97205, Mexico; fhs@cicy.mx (F.H.-S.); hugojoel@cicy.mx (H.J.C.-E.); 4 Unidad de Ingeniería de Tejidos, Terapia Celular y Medicina Regenerativa, Instituto Nacional de Rehabilitación “Luis Guillermo Ibarra Ibarra”, Ciudad de Mexico 14389, Mexico; mvelasquillo@ciencias.unam.mx

**Keywords:** PCL/PLGA, Dental Follicle Mesenchymal Stem Cells (DFMSCs), tissue engineering, articular hyaline cartilage

## Abstract

Articular cartilage is a specialized tissue that provides a smooth surface for joint movement and load transmission. Unfortunately, it has limited regenerative capacity. Tissue engineering, combining different cell types, scaffolds, growth factors, and physical stimulation has become an alternative for repairing and regenerating articular cartilage. Dental Follicle Mesenchymal Stem Cells (DFMSCs) are attractive candidates for cartilage tissue engineering because of their ability to differentiate into chondrocytes, on the other hand, the polymers blend like Polycaprolactone (PCL) and Poly Lactic-co-Glycolic Acid (PLGA) have shown promise given their mechanical properties and biocompatibility. In this work, the physicochemical properties of polymer blends were evaluated by Fourier Transform Infrared Spectroscopy (FTIR) and Scanning Electron Microscope (SEM) and were positive for both techniques. The DFMSCs demonstrated stemness by flow cytometry. The scaffold showed to be a non-toxic effect when we evaluated it with Alamar blue, and the samples were analyzed using SEM and phalloidin staining to evaluate cell adhesion to the scaffold. The synthesis of glycosaminoglycans was positive on the construct in vitro. Finally, the PCL/PLGA scaffold showed a better repair capacity than two commercial compounds, when tested in a chondral defect rat model. These results suggest that the PCL/PLGA (80:20) scaffold may be suitable for applications in the tissue engineering of articular hyaline cartilage.

## 1. Introduction

Articular cartilage is a highly specialized connective tissue of diarthrodial joints, which main function is to provide a smooth and lubricated surface for joint movements and to facilitate the transmission of loads. The hyaline cartilage of the joints has a high-water content but lacks nerves and blood vessels [1] and consequently has a very limited regenerative capacity when an injury occurs [2]. Treatments used for hyaline cartilage injury are aimed to alleviate the pain and include weight control, physical therapy, and pharmacological/surgical treatments, but without permanent results [3]. Moreover, the newly generated tissue formed by the treatments used is fibrocartilage tissue. However, this tissue has been shown to have poor mechanical and structural properties when compared to hyaline cartilage [4]. Therefore, the exploration and investigation of reparative and regenerative alternatives that will result in more physiological and long-lasting results are needed. In this regard, tissue engineering has become an attractive alternative [5,6] since it involves the combined use of different cell types, scaffolds, growth factors, and physical stimulation to regenerate living tissue [7]. Among the most used cells are the mesenchymal stem cells (MSCs), responsible for the maintenance of adult tissues [8]. MSCs have been known for their multipotency [9,10] and can be isolated from several tissues, including oral tissue [11]. MSCs from different sources have differences in growth performance and differentiation potential [12], suggesting a wide range of potential for tissue engineering [13]. Oral-derived MSCs, offer the clinical advantages of easy access, and remarkable tissue reparative/regenerative potential; therefore, have been proposed as ideal candidates for MSCs-based tissue regeneration [14]. Dental Follicle Mesenchymal Stem Cells (DFMSCs) can be isolated from the follicles of the human third molar; these cells present a fibroblast-like morphology. Interestingly, when combined with different scaffolds in different microenvironments, DFMSCs can form a variety of tissues and may differentiate into chondrocytes due to their multipotency, are also adipocyte, osteocytes, neural cells, periodontal, ligament, fibroblast, and hepatocyte-like cell precursors [15,16,17]. Nevertheless, tissue engineering advances and their success depend not only on the proper selection of the cell type; the scaffolding materials selection is also very important. Biomaterials are one of the essential components in making scaffolds; they can be synthetic, natural, or a combination of both. For cartilage tissue engineering, synthetic polymers are diverse and promising, because they are degraded in a regulated manner while the regenerative process of the tissue develops [18]. Polycaprolactone (PCL) and Poly Lactic-co-Glycolic Acid (PLGA) are a kind of biomaterials that have been approved by the FDA for clinical use, and due to their physically strong and highly biocompatible nature, they have been studied as a drug delivery device, suture, or adhesion barrier [19,20]. There are several reports about PCL and PLGA properties, and different proportions have been considered for medical applications, given the impact of their interactions with biological systems [21]. However, relatively few studies have been published with the ratio (80:20), which exhibits a good balance of mechanical and degradation properties, making it promising for tissue engineering. The proportion PCL/PLGA 80:20 has been analyzed in vascular grafts [22], in drug release [23,24], in nerve regeneration [25,26], for skin and osteogenic engineering [27,28,29], and for its general characteristics in tissue engineering [30]. In addition, changes in the diameter and morphology of the fibers as a function of the content of PLGA, as well as different mechanical properties, have been reported by our group [31]. In this work, we probed a PCL/PLGA (80:20) scaffold in combination with cells as an option for tissue engineering of articular hyaline cartilage. Results obtained in vitro and in vivo, using a rat model, suggest that this scaffold may be suitable for future applications in the tissue engineering of articular hyaline cartilage.

## 2. Materials and Methods

### 2.1. Polymer Blends Preparation by Chloroform and Ethanol

Two polymers with the following characteristics were used: PCL (Perstorp Specialty Chemicals, Perstorp, SWE) Mw = 80,000 Da, Tm = 58–60 °C, and Tg = −60 °C, and PLGA (Sigma Aldrich, St. Louis, MO, USA) 75:25 lactic/glycolic, Mw = 76,000–115,000 Da, Tg = 49–55 °C. To obtain PCL/PLGA (80:20) polymers blends were dissolved in a mixture of chloroform/ethanol (J.T. Baker Fisher Scientific, Millersburg, PA, USA) (10:1 *v*/*v*), in agitation at 25 °C for 24 h.

### 2.2. Scaffold Manufacture

For PCL/PLGA (80:20) manufacture, the blend solution (3 mg/11 mL) was collected in a syringe with a stainless-steel needle. The flow rate of the polymer solution was 0.2 mL/h, and the distance between the needle and collector was 13 cm with 20 kV voltage. For the process electrospinning equipment NABOND (model TL-01, Nanotechnology Companies, Nagoya, JPN). The membrane obtained was 10 cm in diameter and had a thickness of approximately 0.16 mm.

### 2.3. Scaffold Characterization

#### 2.3.1. Fourier Transform Infrared Spectroscopy (FTIR)

The identification of functional groups of the PCL/PLGA (80:20) scaffold was performed using Fourier Transform Infrared spectroscopy (FTIR) in the Nicolet equipment (Model 8700, Thermo Scientific, Waltham, MA, USA), with a Zinc selenide (ZnSe) crystal in the 650 cm^−1^ to 4000 cm^−1^ wave number range and averaging 100 scans.

#### 2.3.2. Contact Angle Measurement 

Contact angle measurement is a quantitative way to evaluate whether a given surface has hydrophobic or hydrophilic characteristics. Five microliter drops of distilled water were laid on the PCL/PLGA (80:20) scaffold in the equipment Tantec (Half-Angle, Schaumburg, IL, USA), subsequently, photographs of the drop were captured for 35 seg. The sample was analyzed using a naval research laboratory (NRL) contact angle goniometer by the sessile drop method, placed on each mat, and the contact angle was measured using imaging software (SCA 20).

#### 2.3.3. Differential Scanning Calorimetry (DSC) and Thermogravimetric Analysis (TGA)

The polymer blends’ thermal properties were measured using differential scanning calorimetry in a DSC (Model 7, Perkin-Elmer, Shelton, CT, USA), using a scan rate of 10 °C/min in the temperature interval of 20 °C at 140 °C, with a nitrogen atmosphere. The polymers blend and thermal degradation determinations were performed using thermogravimetric analysis in a TGA (Model 8000, Perkin-Elmer, Shelton, CT, USA), using a scan rate of 10 °C/min in the temperature interval of 50 °C at 700 °C, with a nitrogen atmosphere.

#### 2.3.4. Tensile Mechanical Test 

Dynamic mechanical testing was performed using a Dynamic Mechanical Analyzer (DMA Model 7, Perkin-Elmer, Shelton, CT, USA) in the tensile testing mode. A temperature interval between −20 °C and 80 °C and a scan rate of 3 °C/min were used. The frequency was held constant at 1 Hz, and an initial load of 0.76 × 10^5^ Pa was applied to the sample.

#### 2.3.5. Microstructural Morphology by Scanning Electron Microscope (SEM)

The morphology and microstructure of the electrospun fibers were examined by scanning electron microscope (Model JSM- 7100F, Jeol Ltd., Tokyo, JPN). Samples were previously fixed 2.5% (*v/v*) glutaraldehyde in 0.1 M sodium cacodylate buffer pH 7.2, dehydrated in increasing concentrations of ethanol, dehydrated to a critical point with hexamethyldisilazane (Polysciences, Inc., Warrington, PA, USA) and coated with gold particles under vacuum in an ion sputtering device (JFC 1100, Jeol Ltd., Tokyo, JPN). The Image-J software (Java 8, NIH, Rockville Pike, MD, USA) was used to analyze the captured images. The average diameter of the fibers and pore size were calculated after measuring at least 100 fibers, and the data obtained were subjected to statistical analysis using the GraphPad Prism software version 8 (GraphPad Software, San Diego, CA, USA).

### 2.4. In Vitro Assays

#### 2.4.1. Isolation and Characterization of Dental Follicle Mesenchymal Stem Cells (DFMSCs)

Cells were collected from one volunteer after informed patient consent and ethical approval by the Ethics Committee (Human studies) of the Center for Research and Advanced Studies of the IPN, (number 048/201). The third molar was removed from the patient, and the dental follicle was collected. The cells were released from the tissues by digestion with 1 μg/μL of collagenase type II (Sigma Aldrich St. Louis, MO, USA) for 5 min at 37 °C, and cultured in plastic flasks (TPP, Trasadingen, SH, CHE) containing DMEM Low Glucose medium (Dulbecco’s Modified Eagle Medium; Gibco, Merelbeek, BEL), supplemented with 10% fetal bovine serum (FBS; Gibco, Merelbeek, BEL), and 1% de antibiotic/antifungal (PAA, the cell culture company, Cölbe, DE) (DMEM supplemented medium) and incubated at 37 °C and 5% CO_2_. After 24 h (h), the cells were washed with phosphate-buffered saline (PBS; Biowest, Nuaillé—FRA to remove non-adherent cells, and a fresh DMEM-supplemented medium was added until confluency was reached. Dental follicle Mesenchymal Stem Cells (DFMSCs) from the fourth passage were used in the experiments.

#### 2.4.2. Phenotype Determination by Flow Cytometry

The cells were suspended and incubated in PBS supplemented with 2% FBS (Gibco, Merelbeek, BE) containing monoclonal antibodies to detect CD90 (PC5 conjugated), CD73 (PEC conjugated), CD105 (PC7 conjugated), CD11b (VB610 conjugated), CD14 (APC conjugated), and HLA-DR (APC-A750 conjugated) (BD, Franklin Lakes NJ, USA), all antibodies diluted 1:1000, for 20 min at room temperature (RT). The expression of markers on the cell surface was determined by flow cytometry, performed on a CytoFLEX LX (BRVYNI, Beckman Coulter, Brea, CA, USA). Data were processed using FlowJo software (FlowJo, Ashland, OR, USA).

#### 2.4.3. Phenotype Determination by Immunofluorescence 

The expression of surface markers of cells also was determined by immunofluorescence. Cells were cultured on glass slides for 24 h, washed with PBS, and fixed for 15 min at RT in 3.7% paraformaldehyde (PFA, Spi-Chem, Chemicals—SPI Supplies, West Chester, PA, USA) in PBS. Then cells were permeabilized for 30 min at 4 °C with 3% Triton X-100 (Sigma Aldrich, St. Louis, MO, USA) in PBS, and unspecific sites were blocked for 1 h at RT with 1% BSA (Sigma Aldrich, St. Louis, MO, USA) in PBS. The cells then were incubated overnight at 4 °C with the primary monoclonal antibodies anti-Thy-1 (CD90), CD73, and CD45 (Santa Cruz, Biotechnology, Santa Cruz, CA, USA) diluted 1:40 in blocking solution. Afterward, cells were washed with PBS and incubated with a secondary antibody (FITC-anti-mouse, Jackson InmunoResearch Laboratories Inc., Baltimore, MA, USA) diluted 1:100 in blocking solution at RT for 1 h. Cell nuclei were counterstained with 40,6-diamidino-2-phe-nylindole 1 mg/1 mL (DAPI, Sigma Aldrich, St. Louis, MO, USA). Finally, the slides were washed and coverslipped with VECTASHIELD mounting medium (Vector Laboratories, Inc., Burlingame, CA, US). The slides were observed in a 40× objective epifluorescence microscope ZEISS SCOPE.A1(ZEISS, Oberkochen, Germany) (ZEISS ZEN 3.3, blue edition Software).

#### 2.4.4. Alamar Blue Viability Assay

The cytotoxicity of the PCL/PLGA (80:20) scaffold was determined by the Alamar Blue assay (Thermo Fisher, Pittsburgh, PA, USA), in compliance with the ISO 10993-5, which refers to the in vitro cytotoxicity evaluation of medical devices. DFMSCs (1 × 10^3^) were seeded in triplicate over the PCL/PLGA (80:20) scaffolds, or directly over the plastic as controls, in a 96-well culture dish (TPP, Trasadingen, SH, CHE). The DFMSCs were cultured with DMEM supplemented medium and chondrogenic medium (MesenCult™-ACF Chondrogenic, Differentiation Medium (human), Stem cell technologies, Vancouver, BC, CAN), under standard culture conditions. After 3, 7, 10, 21, and 28 days of culture, the medium was removed, 90 µL of fresh medium and 10 µL of Alamar blue™ were added per well, and the DFMSCs further incubated for 4 h. Alamar blue absorbance was read at 570 nm in a microplate reader (Biotek Elx808, Winooski, VT, USA). Results were normalized with respect to control cells (taken as 100%), seeded in the well without scaffold, and expressed as percentages. 

#### 2.4.5. Adhesion DFMSCs Determined by SEM

DFMSCs (approximately 1 × 10^6^) were seeded on the PCL/PLGA (80:20) scaffold (80:20), maintained in standard culture conditions with supplemented DMEM and evaluated after 4 h, 10, and 28 days. At the indicated times, samples were washed with PBS and fixed with 2.5% (*v*/*v*) glutaraldehyde in 0.1 M sodium cacodylate buffer pH 7.2 for 48 h. Samples were dehydrated in increasing concentrations of ethanol, dehydrated to a critical point with hexamethyldisilazane, and coated with gold particles in an ion-sputtering device. The samples were examined with a field emission scanning electron microscope. To analyze the captured images the Image-J software (Java 8, NIH, Rockville Pike, MD, USA) was used.

#### 2.4.6. Cytoskeleton Organization

DFMSCs (approximately 1 × 10^6^) were seeded on the PCL/PLGA (80:20)scaffold for 4 h, 10, and 28 days then fixed with 3.7% paraformaldehyde (Spi-Chem, Chemicals—SPI Supplies, West Chester, PA, USA) for 15 min at RT, permeabilized with 0.3% Triton X-100 (Sigma Aldrich, St. Louis, MO, USA) and 0.05% TWEEN-20 (Sigma Aldrich, St. Louis, MO, USA) for 30 min and incubated with 0.5% BSA (Sigma Aldrich, St. Louis, MO, USA) in PBS at RT for 10 min. The actin cytoskeleton was visualized using FITC-labeled phalloidin (1:100) (Sigma Aldrich, St. Louis, MO, USA) followed by nuclei staining with DAPI (Sigma Aldrich, St. Louis, MO, USA) and mounting with VECTASHIELD (Vector Laboratories, Inc., Burlingame, CA, USA). Fluorescence images were obtained using a Zeiss confocal microscope (Model LSM700 analyzed with ZEISS ZEN 3.3 (blue edition software).

#### 2.4.7. Quantification of Sulfated Glycosaminoglycans (GAGs)

Sulfated glycosaminoglycan (GAGs) production was evaluated by dimethyl methylene blue (DMB) dye [32]. The DFMSCs (1 × 10^6^) were seeded either directly on the wells, and on the scaffold PCL/PLGA (80:20), or over the commercial hydrogels: Matrigel (Thermo Fisher, Pittsburgh, PA, USA) and TrueGel (Sigma Aldrich, St. Louis, MO, USA), under chondrogenic conditions. At 28 days of induction, all constructs were digested in a buffer containing 100 mM sodium phosphate, 10 mM EDTA, 10 mM L-cysteine, and 0.150 mg/mL papain pH 6.4 for 3 h at 65 °C. In brief, 125 μL of DMB dye solution was added to 40 μL of the digested sample, and the optical density of the solution was read at 595 nm in a microplate reader (Epoch—BioTek, Winooski, VT, USA). The concentration of GAGs was calculated from a standard curve generated with chondroitin 4-sulfate (Sigma Aldrich, St. Louis, MO, USA).

All the experiments were performed at least 3 times, and results were expressed as mean ± SD. Statistical calculations were analyzed by one-way ANOVA, Bonferroni’s multiple comparisons test for all data at a statistical significance level of *p* < 0.05. 

### 2.5. In Vivo Assays 

#### 2.5.1. Articular Cartilage Defects and Scaffolds Implantation

The animals were kept in cages under controlled illumination and temperature, with pellet food and water *ad libitum*. Rats were divided into 5 groups (n = 5); healthy rats, rats with defects, and rats implanted with the PCL/PLGA (80:20) scaffold; in addition, as controls, two groups were implanted with commercial hydrogels, Matrigel and TrueGel. Wistar rats were anesthetized with 5% isoflurane (Fluriso, VetOne, Boise, ID, USA) in oxygen (2.4 L/min), and the right knee of each rat was shaved, and disinfected with benzal solution (Antibenzil, Altamirano, CDMX, MEX). The experimental surgical procedure to perform the chondral defects has been reported in detail elsewhere [33] and entails a unilateral full-thickness articular cartilage defect (2 mm in diameter) in trochlear grooves by carefully drilling, using a 2 mm wide drill 3 mm deep. After removing cartilage debris, the scaffolds, previously carefully trimmed to the size of the defect diameter were implanted to cover the defect in all its thickness; approximately 18 to 20 layers were placed in a dental packer, without exerting pressure, just enough to take it to the defect area. Given that the scaffold slightly distends or expands after being placed in the defect, which allows for its mechanical retention, no fixation elements were required. The surgical site was closed using 4-0 black silk (Futura Surgicare, Bangalore, IND). After the surgery, and during recovery, rats were monitored twice a day, for 5 days to discard discomfort. At 91 days post scaffold implantation, the animals were euthanized by CO_2_ inhalation.

The nature of de novo tissue was assessed by H&E and Alcian blue staining. H&E stain allows the evaluation of tissue repair, integration to the border zone, and structural characteristics, such as cellularity, structural integrity, thickness, and bonding to the adjacent tissue; Alcian blue allowed the evaluation of proteoglycans on the cartilage hyaline matrix.

#### 2.5.2. Histological Analysis

The right knee was dissected, fixed in 3.7% formaldehyde for 48 h at 4 °C decalcified with EDTA 0.5 M (pH 8.0) for 30 days at 4 °C, and cryo-protected in 10% sucrose in PBS for 24 h. Frozen samples of joints were sectioned in the coronal plane of the tissue in the cryostat (Model CM1100, Leica Microsystems, Wetzlar, DEU) to obtain 12 μm thick slices, which were mounted on gelatin-coated slides. Mounted samples were stored at −20 °C until use.

#### 2.5.3. Hematoxylin and Eosin (H&E)

Slides samples were hydrated with PBS, and then stained with Harris hematoxylin (Sigma Aldrich, St. Louis, MO, USA) solution for 10 min, following a standard protocol. Samples were observed under the microscope (Leica DMLS) and photographs with a digital camera Leica DC30 were taken.

#### 2.5.4. Alcian Blue

Slides samples were hydrated with PBS, then stained with Alcian blue according to Alcian Blue Staining Kit instructions (ABlue, ScienCell Research Laboratories, Carlsbad, CA, USA).

## 3. Results

### 3.1. Scaffold Characterization

#### 3.1.1. Fourier Transform Infrared Spectroscopy (FTIR)

**Figure 1:**
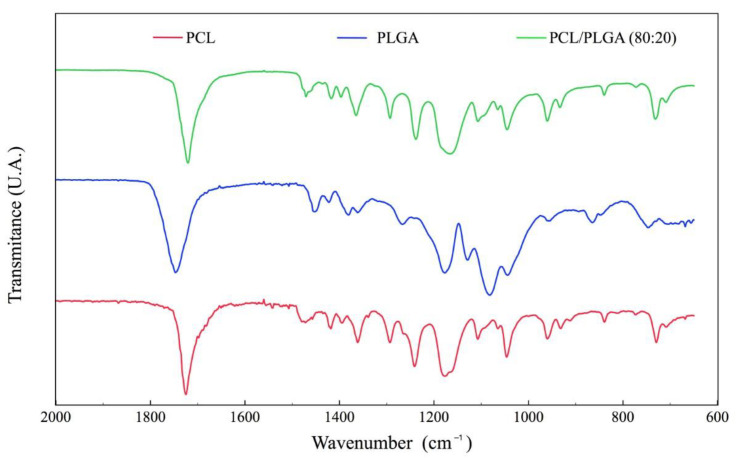
Comparison FTIR spectrum of PCL and PLGA with PCL/PLGA (80:20) scaffold.

Figure 1 shows the spectrum of the signal from the PCL and PLGA polymers. Carbonyl bands were identified at 1733 cm^−1^, and the C-O and C-C groups were also observed at tension at 1300 cm^−1^. At 1244 cm^−1^ the COC peak in asymmetric tension was identified and at 1188 cm^−1^ the OC-O peak in tension. All these functional groups are characteristic of PCL and PLGA, testing the purity of the elements that make up the scaffolding since no contaminants from other materials were observed.

#### 3.1.2. DSC and TGA

**Figure 2:**
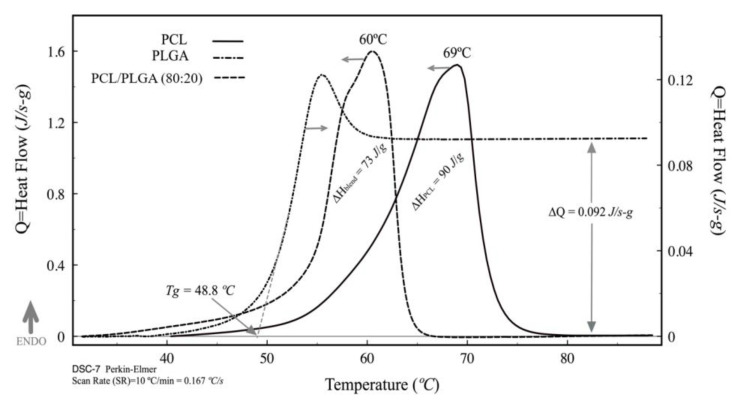
Differential scanning analysis for PCL and PLGA with PCL/PLGA (80:20) scaffold.

DSC thermograms of PCL, PLGA, and the PCL/PLGA (80:20) scaffold are shown in Figure 2. PCL is a semicrystalline polymer, so its thermogram presents a melting peak at 69 °C and presenting a fusion enthalpy of 90 J/g. PLGA is a 100% amorphous polymer; its thermogram presents the glass transition as an abrupt change in heat flow, ∆Q. The glass transition temperature (T_g_) is the onset between the baseline and the transition slope, giving a T_g_ = 48.8 °C. The thermogram of PLGA presents a small peak at the end of the glass transition. This small peak corresponds to structural relaxation (polymer densification), showing the amorphous phase of all polymers under certain temperature conditions [34]. The scaffold thermogram presents the fusion peak of the PCL, and the small “hump” at 58.8°C corresponds to the PLGA glass transition within the blend. The presence of 20% PLGA in the PCL lowers its melting temperature and the heat of fusion (from 60 °C to 69 °C and 90 J/g to 73 J/g) testing the stability of materials after electrospinning.

**Figure 3:**
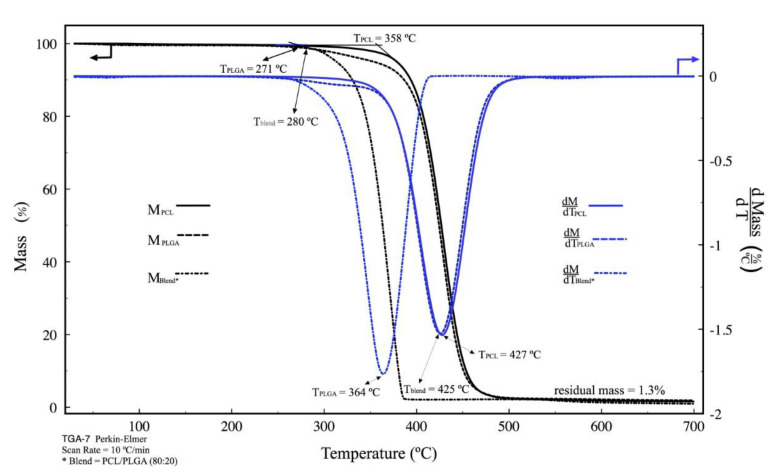
Thermogravimetric analysis for PCL, PLGA, and PCL/PLGA (80:20) scaffold and its derivatives. Black lines correspond to the mass, blue lines correspond to the derivative of the mass respecting the temperature.

Figure 3 shows the TGA thermograms and their derivatives of the PCL, PLGA, and their blend. TGA thermograms showed only one Gaussian peak in each one of the samples, indicating that there was only one decomposition event in the pure polymers and the blend. The PLGA was the first to decompose within the blend (Tblend = 271 °C), which is why the decomposition temperatures of the PLGA and the blend coincided; Tblend = 280 °C and TPLGA = 358 °C. This did not occur with the temperature of maximum decomposition kinetics (pick temperature of derivative) because of the high PCL content within the blend; thus, the temperature of maximum decomposition kinetics is governed by the PCL, Tblend = 425 °C and TPCL = 427 °C.

#### 3.1.3. Evaluation of Wettability by Contact Angle

**Figure 4:**
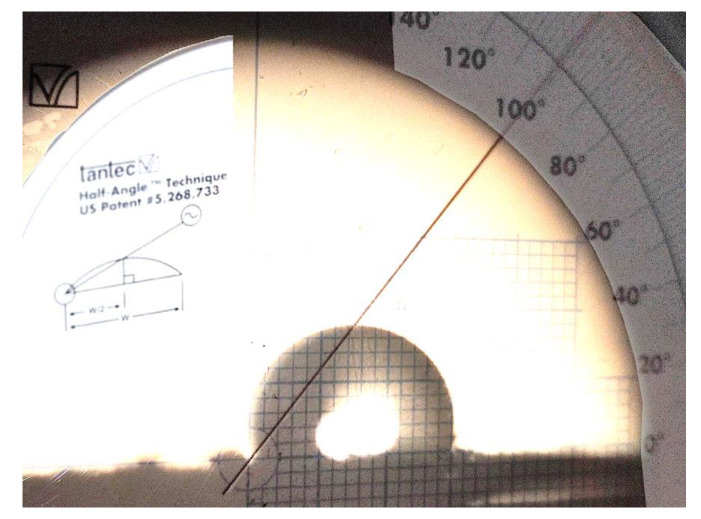
Contact Angle values of PCL/PLGA (80:20). The technique used to measure the contact angle was Half-angle.

Figure 4 shows that the scaffold contact angle made with the PCL/PLGA (80:20) was 100°, in agreement with previous results where the contact angle for PCL [34] and PLGA [35] were 120° and 97°, respectively. The presence of 20% PLGA in the blend lowers the contact angle with respect to PLC by 17%. This indicates that the PLGA’s influence on contact angle in the blend is stronger than for PCL. If the influence were to be equal for the two polymers, the contact angle would have been 115.4° (lineal rule).

When putting the drop of water on the scaffolding, 35 s were allowed to pass before determining the value of the angle. Over time, it was observed that the droplet did not change its dimensions, which means that there is no capillarity effect by the pores that form the microfibers. The contact angles of PCL (120°), PLGA (97°), and the blend (100°) indicate that the three samples present hydrophobic surfaces.

#### 3.1.4. Tensile Mechanical Tests

**Figure 5:**
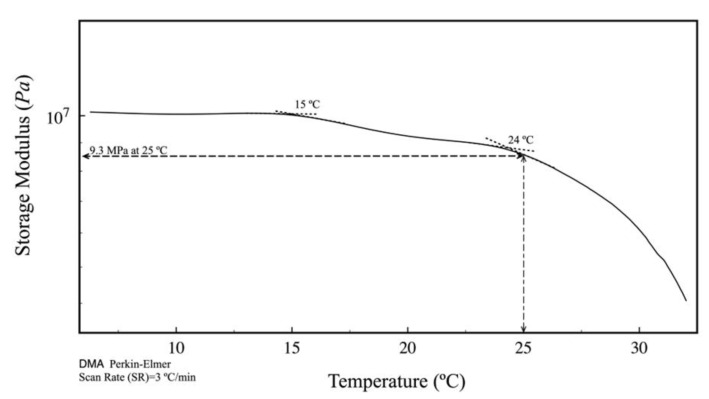
Tensile mechanical test PCL/PLGA (80:20) scaffold.

The storage and dissipation modules of the PCL/PLGA (80:20) scaffold refers to the elastic part modulus of the polymer chains. As the temperature increases, the chains become more mobile, causing a decrease in the storage modulus. Two relaxations were observed in the thermogram (Figure 5), the first at 15 °C, which can be associated with the PLGA glass transition, and the second at 24 °C, which is associated with PCL melting. The storage modulus of the mixture at 25 °C corresponds to the elastic modulus (Young modulus) at the same temperature. The electrospun modulus at 25 °C was 9.3 MPa, DMA of the electrospun blend, storage modulus as a function of temperature.

#### 3.1.5. Microstructural Morphology by Scanning Electron Microscope

**Figure 6:**
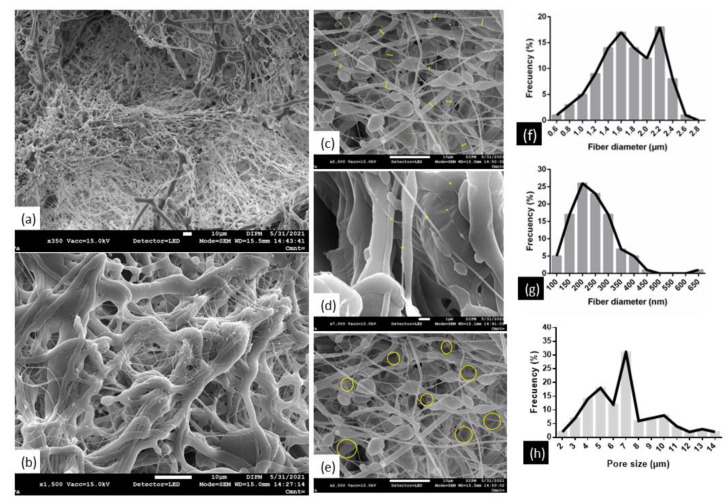
SEM images of PCL/PLGA (80:20) scaffold in different magnifications (**a**,**b**). The analysis of the diameter of the fibers ranging from microns (**c**) to nanometers (**d**), the analysis of the space between the pores (**e**), and the frequency distribution of data obtained for the diameter of the fibers (**f**,**g**) and the pore size (**h**) are shown in the graphs. Yellow circles showed pores.

Fibers in the scaffold PCL/PLGA (80:20) were randomly oriented (Figure 6a), in a greater magnification, thick fibers are also observed, and thin fibers are deposited on them (Figure 6b). To measure the diameter of both fibers (Figure 6c,d) the Image-J software (Java 8, NIH, Rockville Pike, MD, USA) was used to analyze the captured images was used. The analysis of the frequency of distribution showed diameters of thick fibers in 0.6–2.8 µm (Figure 6f) and thin fibers in 100–450 nm (Figure 6g). Pore size was also measured (Figure 6e), with an average pore size of 6.6 µm based on the frequency of distribution (Figure 6h).

### 3.2. In Vitro Assays

#### 3.2.1. Isolation and Identification of DFMSCs by Flow Cytometry and Immunofluorescence 

**Figure 7:**
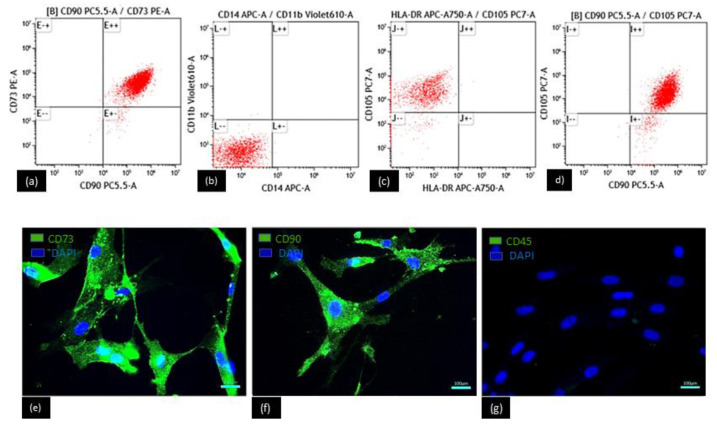
DFMSCs. Expression of surface markers of cells also determined by flow cytometry CD90, CD73, CD105 positive and CD11b, CD14, and HLA-DR negative (**a**–**d**), by immunofluorescence CD73, CD90 positive (in green) and C45 negative (**e**–**g**) done, where only the presence of nuclei is observed (in blue). Scale bar = 100 µm.

MSC isolated from dental follicles were characterized by the detection of several specific antigens by flow cytometry. The dot plots (Figure 7) showed that DFMSCs were positive for CD73, CD90, and CD105 and CD11b, CD14, and HLA-DR markers (Figure 7a–d) Also, by immunofluorescence we confirmed positive expression of CD73 (Figure 7e) and CD 90 (Figure 7f), while the CD45 marker remained negative (Figure 7g). In addition, most of the isolated cells had a spindle-like shape, elongated, and flattened with characteristics of MSC. Our results indicated the effective purification of MSC for dental follicles since the minimum criteria established by the International Society for Cell & Gene Therapy (ISCT) were fulfilled.

#### 3.2.2. Alamar Blue Viability Assay

**Figure 8:**
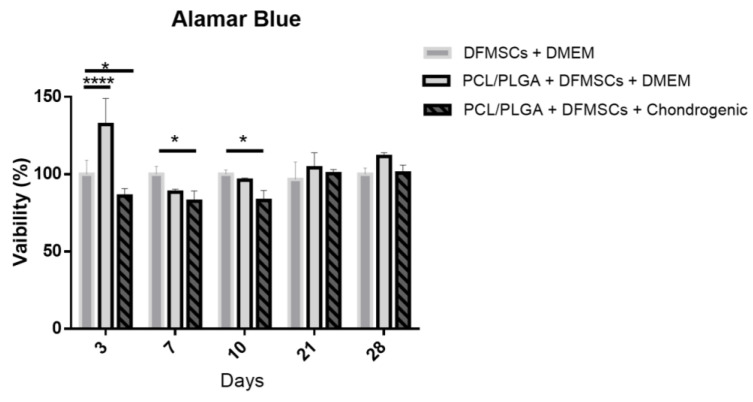
Cellular viability was determined by Alamar Blue. The different bars show DFMSCs grown directly in the well, DFMSCs grown on PCL/PLGA (80:20) scaffold in DMEM complete medium, and in chondrogenic medium (induction). PCL/PLGA: PCL/PLGA (80:20). (ANOVA test * *p* < 0.05, **** *p* < 0.0001).

After 3 days on culture with DMEM medium the DFMSCs grown on the PCL/PLGA (80:20) scaffolds showed an increase in cell viability (132.64 ± 16.85) with respect to control (DFMSCs seeded in the wells); however, DFMSCs on the PCL/PLGA (80:20) under chondrogenic medium showed a decrease in viability at 3 days (85.96 ± 3.78) with respect to control DFMSCs. At 7 and 10 days under chondrogenic medium DFMSCs on the PCL/PLGA (80:20) showed a statistical difference (82.57 ± 6.67) (83.083 ± 6.4) respectively, compared with the control DFMSCs in the well. Finally, at 21 and 28 days evaluated, no statistical difference between groups was observed. The viability results are shown in Figure 8.

#### 3.2.3. Adhesion DFMSCs by SEM

**Figure 9:**
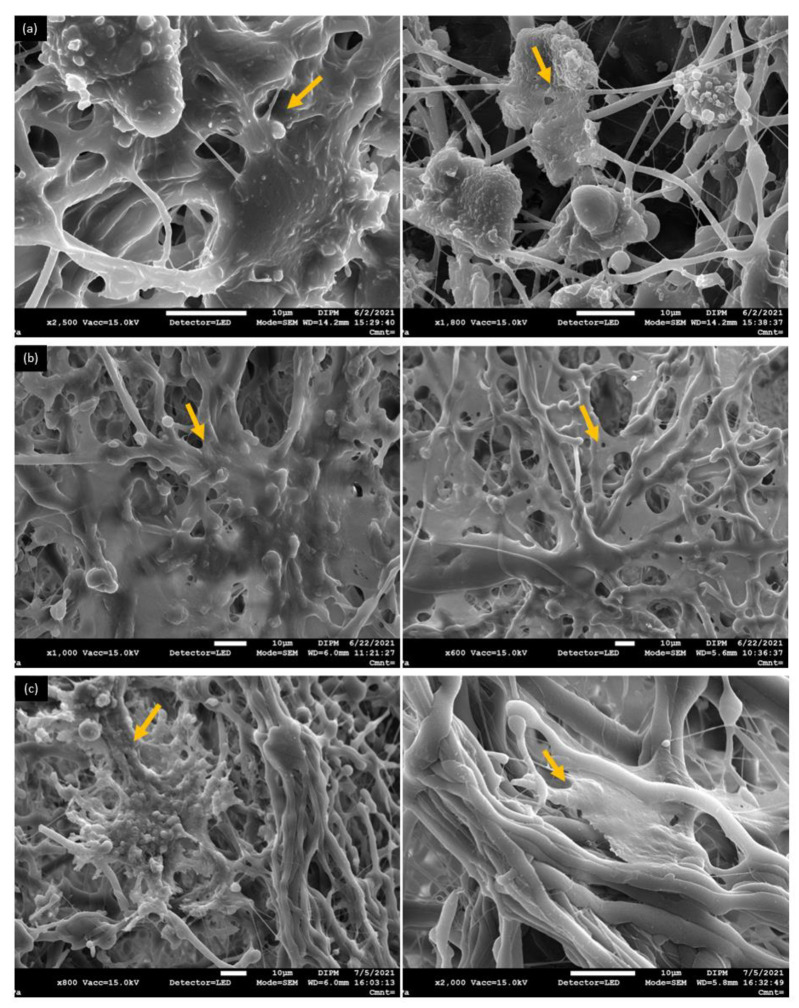
SEM images of DFMSCs grown on PCL/PLGA (80:20) scaffold after 4 h (**a**), 10 days (**b**), and 28 days (**c**). DFMSCs are arrowed in yellow.

SEM was used to determine the adhesion and morphology of the DFMSCs cultured on the PCL/PLGA (80:20) in a chondrogenic medium. In the images at 4 h (Figure 9a), the DFMSCs had a direct interaction with fibers on the PCL/PLGA (80:20) scaffold. At 10 days (Figure 9b), DFMSCs were observed in the deep layers, suggesting migration through the fibers of the scaffold and therefore, the formation of a three-dimensional and multicellular network according to the scaffold architecture. At 28 days (Figure 9c) DFMSCs spread on the fibers and formed a monolayer that covered large areas of the scaffold. Our results from microscopy images suggest that the PCL/PLGA (80:20) scaffolds are cytocompatible and cell-friendly in in vitro settings.

#### 3.2.4. Cytoskeleton Organization

**Figure 10:**
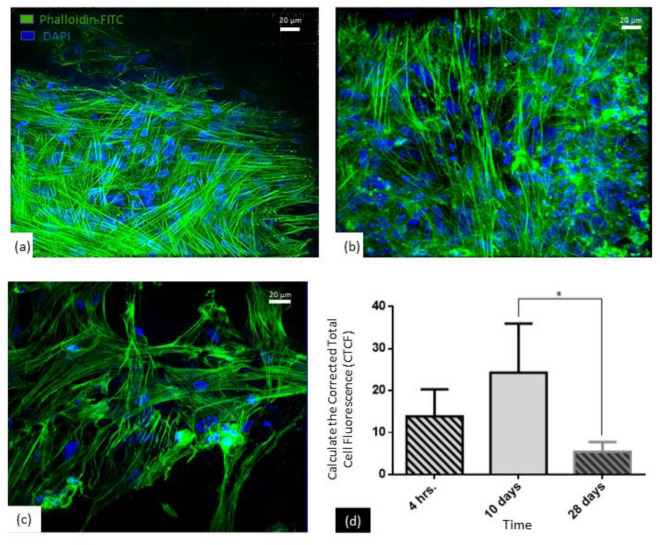
Confocal microscopy analysis of DFMSCs grown on PCL/PLGA (80:20) scaffold after 4 h (**a**) 10 (**b**), and 28 days (**c**). F-actin is shown in green and nuclei in blue. Scale bar = 20 µm. (**d**) quantified F-actin expression per cell; Z-stacks were flattened by integration. (ANOVA test * *p* < 0.05.).

To investigate the influence of the PCL/PLGA (80:20) scaffold on DFMSCs cytoskeleton morphology, the polymerized actin was visualized by phalloidin-FITC staining. The staining also allowed us to determine the integration between DFMSCs and the scaffold (Supplementary Material Video). Into the scaffolds, the DFMSCs cytoskeletal organization appeared to be stretched in a stellate isotropic configuration, characteristic of undifferentiated MSCs, at all times evaluated (Figure 10a–c). In the first 4 h (Figure 10a) and the 10 days (Figure 10b) of analysis, cells expressed a high level of F-actin with respect to 28 days (Figure 10c), which is suggestive that the PCL/PLGA (80:20) scaffold is cell-friendly. However, a significant drop in the level of fluorescence was observed after 28 days of culture (Figure 10d).

#### 3.2.5. Quantitation of Sulfated Glycosaminoglycans

**Figure 11:**
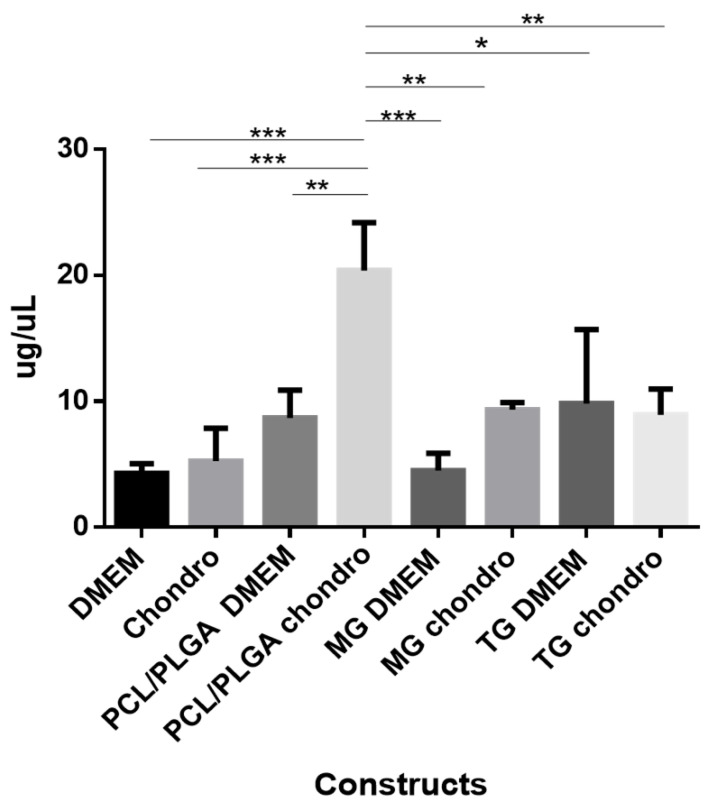
Sulfated glycosaminoglycans (GAGs) quantification after 28 days of chondrogenic differentiation. The different bars show DFMSCs grown directly in the well under DMEM medium and chondrogenic medium (induction); DFMSCs on the different scaffolds; PCL/PLGA (80:20); commercial hydrogels Matrigel and TrueGel, under DMEM medium and chondrogenic medium (induction) (ANOVA * *p* < 0.05, ** *p* < 0.01, *** *p* < 0.001. Constructs: Scaffold + DFMSCs + Medium components. Scaffolds: PCL/PLGA: PCL/PLGA (80:20), MG: Matrigel. TG: TrueGel. Chondro: Chondrogenic medium.

Our results showed that the highest concentration of GAGs was produced by the group of DFMSCs seeded on PCL/PLGA (80:20) scaffold and maintained with chondrogenic medium when compared to DFMSCs seeded on PCL/PLGA (80:20) scaffold cultured in DMEM medium. DFMSCs seeded in the scaffold under chondrogenic conditions showed significantly higher GAGs production than the cells grown in the commercially available scaffolds Matrigel and TrueGel, either in the presence or absence of chondrogenic medium (Figure 11) suggesting the correct differentiation of DFMSCs to chondrocytes.

### 3.3. In Vivo Assay

#### Histology Evaluation

**Figure 12:**
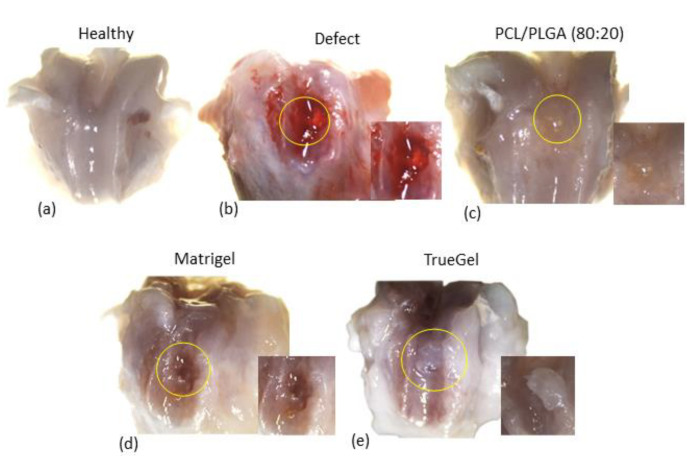
Macroscope analysis of cartilage defects. In the healthy group (**a**), the tissue is observed to be intact and bright, characteristic of hyaline cartilage. The defect group presents evident tissue damage (**b**). In the rats that were implanted with the PCL/PLGA (80:20) scaffold (**c**), a repair tissue is observed that covers the defect. In the rats where the Matrigel (**d**) and TrueGel (**e**) hydrogels were implanted, the edges of the defect were well-defined and with brown coloration.

Rat tissues did not show any signs of infection such as inflammation or extensive fibrosis, after 91 days post-surgery. Lesions in the control rats with defects did not heal (Figure 12b), as shown by the damage at the macroscope levels. Meanwhile, rats implanted with PCL/PLGA (80:20) scaffold showed a reparation of all defects (Figure 12c); for example, the border regions between repaired and normal tissue were less apparent than in other groups and similar to the group of healthy cartilage (Figure 12a). Groups implanted with hydrogels scaffold generated a new tissue but with an evident border region between repaired and adjacent tissue. In the Matrigel group, the defect was covered by a thin layer of fibrous tissue with a well-defined border and evident brown coloration (Figure 12d), while in the TrueGel group, the defect was repaired with a hyaline tissue, which easily detaches from the area (Figure 12e).

**Figure 13:**
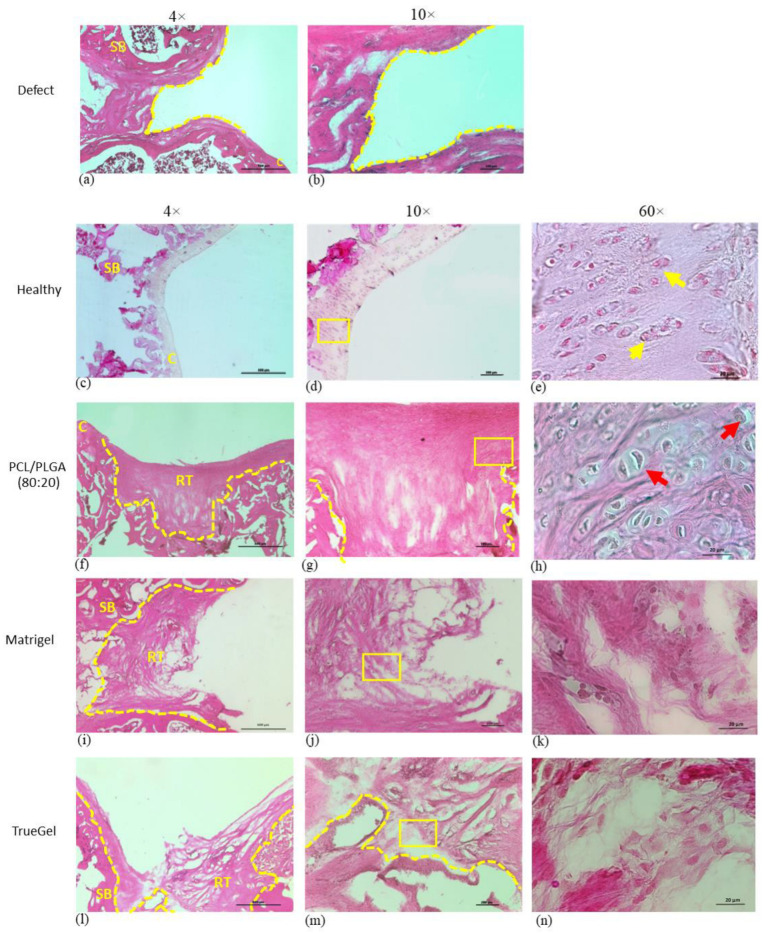
Repaired tissue of cartilage treated with different scaffolds. Tissue sections were stained with H&E and visualized by light microscopy. Without a scaffold, the defect remained open (**a**,**b**). In the healthy group (**c**–**e**), the tissue has an architecture characteristic of hyaline cartilage. In the rats that were implanted with the PCL/PLGA (80:20) scaffold whole area defect was covered with proper integration into the adjacent cartilage (**f**,**g**), with chondrocytes-like cells (**h**). Defects in the rats implanted with Matrigel (**i**–**k**) and TrueGel (**l**–**n**) hydrogels, the repaired tissue shows a disorganized architecture and fibrous aspect. RT: repaired tissue, C: cartilage, SB: subchondral bone. Dotted lines in yellow indicate the defect area (4×) and the yellow boxes in 10× show the magnified area in 60×. Yellow arrows show chondrocytes and red arrows show chondrocytes-like cells.

Tissues stained with H&E stain and analyzed 91 days post-surgery, clearly showed that the defect in the group without scaffold remained open (Figure 13a,b). Meanwhile, in the group treated with the PCL/PLGA (80:20) scaffold, the presence of chondrocyte-like cells was observed in the repair tissue, with proper integration into the adjacent cartilage (Figure 13f–h). In the Matrigel and TrueGel groups, the articular surfaces in the defect site were fibrous and easily detachable from the cartilage, leaving a defect hole that resembles the untreated group, with a significant gap on the defect (Figure 13i,l). Again, neither inflammatory response nor necrotic tissue associated with tissue damage or granulation was observed.

**Figure 14:**
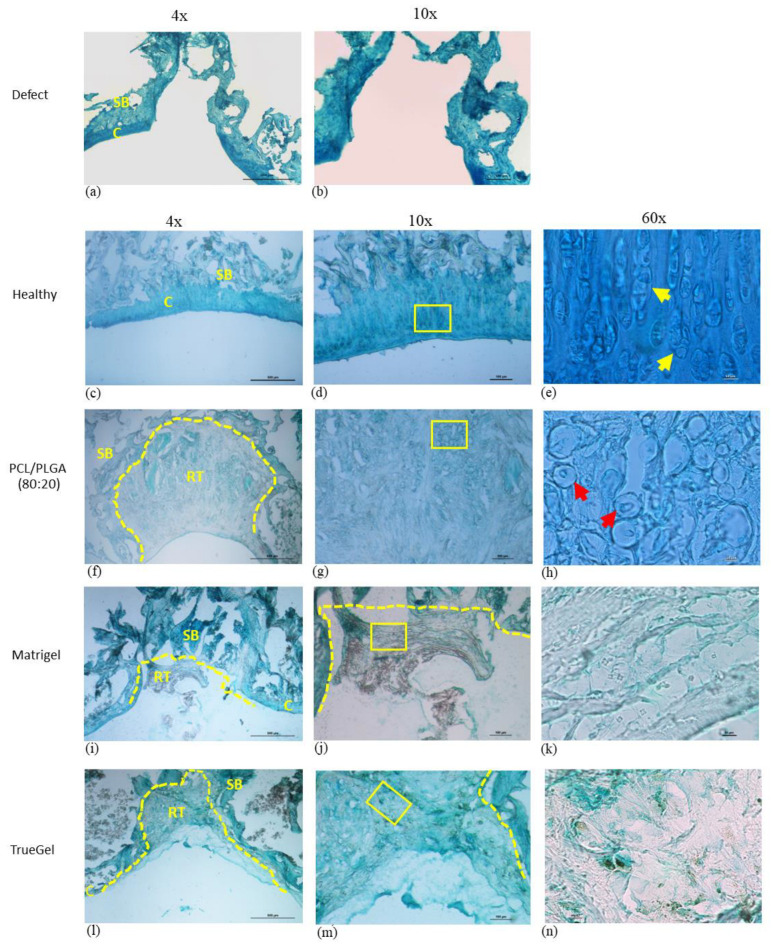
Repaired tissue of cartilage treated with different scaffolds and stained with Alcian blue. The defect did not close where the scaffold was not implanted (**a**,**b**). In the healthy group, the staining is observed uniformly (**c**–**e**). In the group where the PCL/PLGA (80:20) was implanted, the whole defect area is observed filled and with proper tissue integration into the adjacent cartilage (**f**–**h**), with strong staining (**e**). In the group implanted with Matrigel, the tissue is poorly stained (**i**–**k**), and in the group with TrueGel the tissue is stained but without organization (**l**–**n**). RT: repaired tissue, C: cartilage, SB: subchondral bone. Dotted lines in yellow indicate the defect area, and the yellow boxes in 10× show the magnified area in 60×. Yellow arrows show chondrocytes and red arrows show chondrocyte-like cells.

The histological sections of the group of healthy rats stained by Alcian blue showed intense staining in the superficial zone, and somewhat less intense in the peripheral and intermediate that suggested a strong expression of GAGs (Figure 14c–e). Staining on the tissue implanted with the PCL/PLGA (80:20) was homogeneous and the presence of round cells exhibiting the morphology of chondrocytes, and extracellular matrix was observed, furthermore, the defects were filled with repaired thick tissue (Figure 14f–h). In the defects repaired with Matrigel scaffolds, very few cells, and poor stains with Alcian blue were observed; additionally, tissue appeared thin and with detachments in the defect area (Figure 14i,k). Finally, the defects repaired with TrueGel showed intense stained areas alternating with areas with little staining, and few cells (Figure 14l–n).

## 4. Discussion

Biomaterials of different origins have been used to manufacture membranes, films, or scaffolds for the correction of degenerative problems in hyaline cartilages [18]. 

PCL and PLGA biomaterials have been used for medical applications, due to the impact this combination has on their interaction with biological systems [21]. Polymer PCL is characterized by its low cost, good stability, and mechanical strength; however, it has poor hydrophilicity and slow biodegradation [30,36,37]. On the other hand, PLGA has good biocompatibility, and an adequate degradation rate, but has poor mechanical properties [19]. Thus, PCL and PLGA polymers are combined to obtain biocompatible scaffolds showing properties not attainable by the use of any of the constituents alone [26,36]. The ratio of the combined polymers is key to achieving a scaffold with ideal characteristics for cell establishment, proliferation, and differentiation. 

Although there are several published studies on PCL and PLGA, mainly on tissue engineering [25,26,28,29,30] and drug delivery [24,36,38] few have tested the combination (80:20) for cartilage repair.

Therefore, based on this paucity of information and in our previous work [32] we decided to test PCL/PLGA (80:20) scaffolds for chondral defect reparation with potential application in hyaline cartilage tissue engineering. Interestingly, we found that the temperatures obtained in the DMA analysis (Figure 5) do not correspond to those determined in the DSC thermogram (Figure 2). These differences are mainly due to the fact that in the DMA, the electrospun is under static and dynamic stress, while in the DSC, the electrospun is static; however, DMA results coincide in order of magnitude with several authors [23,39]. These data are very important in the design of scaffolds because they give quality control and reproducibility.

In our results, the contact angles of PCL (120°), PLGA (97°), and the blend (100°) indicate that the three samples present hydrophobic surfaces. Lee and Hiep [30] studied a PCL/PLGA scaffold by electrospinning because they are easy to handle and to “play” with the concentrations to reduce hydrophobicity, and it is also possible to regulate or control the degradation times [40,41]. In addition, this technique allows a characterization of the changes in wetting properties of the surfaces through surface modifications if necessary.

Caixia Peng et al. [42] made scaffolds of PCL/PLGA in different proportions (1:90, 50:50, 90:10) reporting good degradation properties, surface characteristics, and cellular activities, demonstrating that the PCL/PLGA (50:50) possessed potential in tissue engineering. These concentrations are very similar to those described by Sanchez-Pech [31] that, demonstrated that scaffolds made by electrospinning of PCL and PLGA (70:30) were not toxic, and the pore sizes and distances between fibers allowed the migration and proliferation of urethral cells, concluding that the mixture with these percentages is recommended for urethral tissue engineering, as it facilitates the turnover of nutrients and oxygen, an important characteristics for proper tissue repair and regeneration. The evaluation of the PCL/PLGA (80:20) scaffold by SEM (Figure 6) shows results that fully agree with what was previously reported by Sanchez-Pech [31] regarding the pore size and diameter of the fibers.

Critchley et al. [43] reported that hydrogel scaffolds could be mechanically reinforced using PCL/PLGA in different proportions (65:35) (85:15); thus becoming capable of supporting robust chondrogenesis. On the other hand, Zamanlui et al. [44] used PCL/PLGA (50:50 and 70:30) scaffolds which showed good properties to go along with chondrogenesis; in their experiments, the authors showed high expression levels of chondrogenic markers such as type II collagen and aggrecan, and conclude that the PCL/PLGA (70:30) scaffold enhances the differentiation to chondrocytes. These variations in the percentages scaffold shall be studied and compared with our scaffold to evaluate the conditions in which they worked and their chondrogenic potential.

The International Standard ISO 10993 (Biological Evaluation of Medical Devices) recommends that materials used in humans must be subjected to in vitro and biocompatibility assays to verify the response of cells interacting with them [45]. According to different studies, the viability that allows the PCL and PLGA scaffolds in isolation and together (PCL/PLGA) is favorable, indicating that they have low toxicity and adequate interaction with biological systems [22,46,47]. Our results showed no cytotoxic effects in the groups evaluated (PCL/PLGA scaffold + DFMSCs cultured in DMEM and PCL/PLGA scaffold + DFMSCs cultured with chondrogenic medium) even on 21 and 28 days, with increased activity of cell metabolism, which translates into greater viability and proliferation above 100% (Figure 2). It has been reported that percentages of 10% and 20% of PLGA with PCL produce nano-porous structures on PCL domains, providing a surface that favors the deposition of proteins and biomolecules enhancing cell adhesion and growth. Yet, PLGA levels higher than 30% generate unfavorable results [29]. 

The sensitivity to the ratio of both polymers can be explained by the fact that during scaffold processing by electrospinning, the morphology of the PLGA/PCL fibers is affected by the percentage of the components. In this sense, it has been described that increasing the amount of PLGA modifies the average diameter of the fibers and their distribution; for example, Sanchez-Pech et al., in2020 reported a reduction in fiber diameter starting at added 10% 20%, and 30% of PLGA in the PCL matrix and pore size decreases when the PLGA content increases [31].

Moreover, PCL/PLGA (80:20) scaffold allows proper cell attachment, as has been observed by SEM and phalloidin staining by immunofluorescence. SEM showed the interaction of the cells with the scaffold and allowed us to observe the cell-material interface, although did not determine any specific adhesion molecules (Figure 9). In turn, the visualization of the cytoskeleton organization by immunofluorescence (Figure 10), not only corroborated the SEM observations but showed cell shape adaptation to the material in response to specific cell point contacts and the migration of the cells deep into the scaffold. The DFMSCs spread and distributed over the fibers, and it was determined that there are changes in the cytoskeleton as the culture time passes, but the cells remain in the whole structure [22]. The surface architecture of the scaffold may stimulate the attachment and facilitate adhesion of the cells, and by signal transduction regulate the cytoskeleton and matrix binding [48,49]. Therefore, the morphology of cells that adhered to the scaffold was visualized to study the modification of the cytoskeleton at different in vitro culture times (supplementary material video). F-actin expression and morphology depend on the establishment of a mechanical force balance in the cytoskeleton [50], and could be associated with tensional forces that are generated within the ECM resulting in mechanical stabilization of the cell shape [51]. Cells sense and respond to the physical properties of the matrix by converting mechanical cues into intracellular chemical signals, which in turn, control gene expression, protein production, and phenotypic behavior [52].

In this regard, although PCL is hydrophobic, PLGA confers certain hydrophilicity and thus, the ability to interact with the cells [26,27,36]. Moreover, the hydrophilicity of the scaffold depends not only on its surface chemistry but also on its roughness and architecture [29,36,53,54,55]. In this sense, the PCL/PLGA (80:20) scaffold showed to be highly porous, according to the SEM technique (Figure 6), allowing anchorage and migration of these cells throughout the thickness of the scaffold. In addition to hydrophobicity and hydrophilicity, other characteristics such as surface energy (tension) have implications for biological responses such as adhesion [53]. Other studies evidenced that the microenvironment around the scaffold was remodeled with newly formed ECM proteins, thus becoming a suitable environment for the proliferation and differentiation of chondrocytes and MSCs [56,57,58].

Quantification of GAGs was higher in the PCL/PLGA (80:20) scaffold in comparison with the DFMSCs grown directly in the well and against different types of scaffolds (Figure 11). Under in vitro conditions, the PCL/PLGA scaffold (80:20) after 28 days of culture is observed to barely lose their integrity but, the Matrigel and TrueGel hydrogels lost their integrity after 15 days of culture. The differences between the DMEM medium and the chondrogenic medium were also evident; as the molecules in the chondrogenic medium did increase the production of GAGs in the PCL/PLGA (80:20) scaffold. Thus, the triad of tissue engineering namely cells, inducer molecules, and scaffolds all work together to modulate the appropriate environment in the tissues to be repaired or regenerated. 

The in vivo studies carried out in a rat model with the PCL/PLGA (80:20) show a qualitative organization similar to samples obtained from the healthy rat group. In contrast, the rapid degradation observed with the Matrigel and TrueGel scaffolds resulted in inadequate mechanical support to the joint tissue, and a poor reparative effect. Moreover, in the Alcian Blue analysis, the PCL/PLGA (80:20) scaffold showed a better structure and stain, the defect was repaired completely, and the presence of chondrocyte-like cells was observed (Figure 14f–h).

In summary, the tested scaffold proved to be non-cytotoxic, and although it presents hydrophobic surfaces, it still favors cellular adhesion and chondrogenic differentiation capacity under in vitro conditions; when tested in a rat in vivo model, the scaffold showed biocompatibility, non- immunogenicity, and good integration into the cartilage. These results taken together are encouraging and suggest that the PCL/PLGA (80:20) scaffold may be suitable for future applications in tissue engineering of articular hyaline cartilage and it can be an option in clinical treatment with better results than currently available treatments.

## Data Availability

Data presented in this study are available upon request to the corresponding author. The data are not publicly available because they involve confidential information due to the novel and multidisciplinary approach in which this work was carried out.

## Supplementary Materials

The following supporting information can be downloaded at: https://www.mdpi.com/article/10.3390/polym15102324/s1, Cytoskeleton Organization: Confocal Z-stack video. The scaffold presents depth, the DFMSCs are observed between the fibers and migrated to deep strata and by the cytoskeleton stain observed that they continue interacting with each other at different culture times. 4 h (Video S1), 10 days (Video S2) and 28 days (Video S3).

## References

[1] Salgado A.J., Oliveira J.M., Martins A., Teixeira F.G., Silva N.A., Neves N.M., Sousa N., Reis R.L. (2013). Tissue Engineering and Regenerative Medicine: Past, Present, and Future. Int. Rev. Neurobiol..

[2] Lolli A., Colella F., De Bari C., van Osch G.J.V.M. (2019). Targeting Anti-Chondrogenic Factors for the Stimulation of Chondrogenesis: A New Paradigm in Cartilage Repair. J. Orthop. Res..

[3] Hunter C.W., Deer T.R., Jones M.R., Chang Chien G.C., D’Souza R.S., Davis T., Eldon E.R., Esposito M.F., Goree J.H., Hewan-Lowe L. (2022). Consensus Guidelines on Interventional Therapies for Knee Pain (STEP Guidelines) from the American Society of Pain and Neuroscience. J. Pain Res..

[4] Patel J.M., Dunn M.G., Nukavarapu S.P., Freeman J.W., Laurencin C.T. (2015). 6-Cartilage Tissue Engineering. Regenerative Engineering of Musculoskeletal Tissues and Interfaces.

[5] Morouço P., Fernandes C., Santos-Rocha R. (2019). Osteoarthritis, Exercise, and Tissue Engineering: A Stimulating Triad for Health Professionals. J. Aging Res..

[6] Stampoultzis T., Karami P., Pioletti D.P. (2021). Thoughts on Cartilage Tissue Engineering: A 21st Century Perspective. Curr. Res. Transl. Med..

[7] Francis S.L., Di Bella C., Wallace G.G., Choong P.F.M. (2018). Cartilage Tissue Engineering Using Stem Cells and Bioprinting Technology—Barriers to Clinical Translation. Front. Surg..

[8] Pekovic V., Hutchison C.J. (2008). Adult Stem Cell Maintenance and Tissue Regeneration in the Ageing Context: The Role for A-Type Lamins as Intrinsic Modulators of Ageing in Adult Stem Cells and Their Niches. J. Anat..

[9] Pittenger M.F., Mackay A.M., Beck S.C., Jaiswal R.K., Douglas R., Mosca J.D., Moorman M.A., Simonetti D.W., Craig S., Marshak D.R. (1999). Multilineage Potential of Adult Human Mesenchymal Stem Cells. Science.

[10] Pittenger M.F., Discher D.E., Péault B.M., Phinney D.G., Hare J.M., Caplan A.I. (2019). Mesenchymal Stem Cell Perspective: Cell Biology to Clinical Progress. npj Regen. Med..

[11] Li B., Ouchi T., Cao Y., Zhao Z., Men Y. (2021). Dental-Derived Mesenchymal Stem Cells: State of the Art. Front. Cell Dev. Biol..

[12] Zhan X.-S., El-Ashram S., Luo D.-Z., Luo H.-N., Wang B.-Y., Chen S.-F., Bai Y.-S., Chen Z.-S., Liu C.-Y., Ji H.-Q. (2019). A Comparative Study of Biological Characteristics and Transcriptome Profiles of Mesenchymal Stem Cells from Different Canine Tissues. Int. J. Mol. Sci..

[13] Wegmeyer H., Bröske A.-M., Leddin M., Kuentzer K., Nisslbeck A.K., Hupfeld J., Wiechmann K., Kuhlen J., von Schwerin C., Stein C. (2013). Mesenchymal Stromal Cell Characteristics Vary Depending on Their Origin. Stem Cells Dev..

[14] Zhou L., Liu W., Wu Y., Sun W., Dörfer C.E., Fawzy El-Sayed K.M. (2020). Oral Mesenchymal Stem/Progenitor Cells: The Immunomodulatory Masters. Stem Cells Int..

[15] Mosaddad S.A., Rasoolzade B., Namanloo R.A., Azarpira N., Dortaj H. (2022). Stem Cells and Common Biomaterials in Dentistry: A Review Study. J. Mater. Sci. Mater. Med..

[16] Chen G., Chen J., Yang B., Li L., Luo X., Zhang X., Feng L., Jiang Z., Yu M., Guo W. (2015). Combination of Aligned PLGA/Gelatin Electrospun Sheets, Native Dental Pulp Extracellular Matrix and Treated Dentin Matrix as Substrates for Tooth Root Regeneration. Biomaterials.

[17] Xu Q.L., Furuhashi A., Zhang Q.Z., Jiang C.M., Chang T.-H., Le A.D. (2017). Induction of Salivary Gland-Like Cells from Dental Follicle Epithelial Cells. J. Dent. Res..

[18] Wasyłeczko M., Sikorska W., Chwojnowski A. (2020). Review of Synthetic and Hybrid Scaffolds in Cartilage Tissue Engineering. Membranes.

[19] Makadia H.K., Siegel S.J. (2011). Poly Lactic-Co-Glycolic Acid (PLGA) as Biodegradable Controlled Drug Delivery Carrier. Polymers.

[20] Elmowafy E.M., Tiboni M., Soliman M.E. (2019). Biocompatibility, Biodegradation and Biomedical Applications of Poly(Lactic Acid)/Poly(Lactic-Co-Glycolic Acid) Micro and Nanoparticles. J. Pharm. Investig..

[21] Zhao W., Li J., Jin K., Liu W., Qiu X., Li C. (2016). Fabrication of Functional PLGA-Based Electrospun Scaffolds and Their Applications in Biomedical Engineering. Mater. Sci. Eng. C Mater. Biol. Appl..

[22] Gao Y., Callanan A. (2021). Influence of Surface Topography on PCL Electrospun Scaffolds for Liver Tissue Engineering. J. Mater. Chem. B.

[23] Chou S.-F., Woodrow K.A. (2017). Relationships between Mechanical Properties and Drug Release from Electrospun Fibers of PCL and PLGA Blends. J. Mech. Behav. Biomed. Mater..

[24] Tiwari S.K., Tzezana R., Zussman E., Venkatraman S.S. (2010). Optimizing Partition-Controlled Drug Release from Electrospun Core–Shell Fibers. Int. J. Pharm..

[25] Alvim Valente C., Cesar Chagastelles P., Fontana Nicoletti N., Ramos Garcez G., Sgarioni B., Herrmann F., Pesenatto G., Goldani E., Zanini M.L., Campos M.M. (2018). Design and Optimization of Biocompatible Polycaprolactone/Poly(l-Lactic-*Co*-Glycolic Acid) Scaffolds with and without Microgrooves for Tissue Engineering Applications: Design and Optimization of Biocompatible PCL/PLGA Scaffolds. J. Biomed. Mater. Res..

[26] Ferreira C.L., Valente C.A., Zanini M.L., Sgarioni B., Ferreira Tondo P.H., Chagastelles P.C., Braga J., Campos M.M., Malmonge J.A., de Souza Basso N.R. (2019). Biocompatible PCL/PLGA/Polypyrrole Composites for Regenerating Nerves. Macromol. Symp..

[27] Franco R.A., Nguyen T.H., Lee B.-T. (2011). Preparation and Characterization of Electrospun PCL/PLGA Membranes and Chitosan/Gelatin Hydrogels for Skin Bioengineering Applications. J. Mater. Sci. Mater. Med..

[28] Tang Z.G., Callaghan J.T., Hunt J.A. (2005). The Physical Properties and Response of Osteoblasts to Solution Cast Films of PLGA Doped Polycaprolactone. Biomaterials.

[29] Tang Z., Hunt J. (2006). The Effect of PLGA Doping of Polycaprolactone Films on the Control of Osteoblast Adhesion and Proliferation in Vitro. Biomaterials.

[30] Hiep N.T., Lee B.-T. (2010). Electro-Spinning of PLGA/PCL Blends for Tissue Engineering and Their Biocompatibility. J. Mater. Sci. Mater. Med..

[31] Sánchez-Pech J.C., Rosales-Ibáñes R., Cauich-Rodriguez J.V., Carrillo-Escalante H.J., Rodríguez-Navarrete A., Avila-Ortega A., Hernández-Sánchez F. (2020). Design, Synthesis, Characterization, and Cytotoxicity of PCL/PLGA Scaffolds through Plasma Treatment in the Presence of Pyrrole for Possible Use in Urethral Tissue Engineering. J. Biomater. Appl..

[32] Stone J.E., Akhtar N., Botchway S., Pennock C.A. (1994). Interaction of 1,9-Dimethylmethylene Blue with Glycosaminoglycans. Ann. Clin. Biochem..

[33] Chung J.Y., Song M., Ha C.-W., Kim J.-A., Lee C.-H., Park Y.-B. (2014). Comparison of Articular Cartilage Repair with Different Hydrogel-Human Umbilical Cord Blood-Derived Mesenchymal Stem Cell Composites in a Rat Model. Stem Cell Res. Ther..

[34] Narayanan G., Shen J., Boy R., Gupta B.S., Tonelli A.E. (2018). Aliphatic Polyester Nanofibers Functionalized with Cyclodextrins and Cyclodextrin-Guest Inclusion Complexes. Polymers.

[35] Paragkumar N.T., Edith D., Six J.-L. (2006). Surface Characteristics of PLA and PLGA Films. Appl. Surf. Sci..

[36] Gao J., Chen S., Tang D., Jiang L., Shi J., Wang S. (2019). Mechanical Properties and Degradability of Electrospun PCL/PLGA Blended Scaffolds as Vascular Grafts. Trans. Tianjin Univ..

[37] Sun H., Mei L., Song C., Cui X., Wang P. (2006). The in Vivo Degradation, Absorption and Excretion of PCL-Based Implant. Biomaterials.

[38] Chou S.-F., Carson D., Woodrow K.A. (2015). Current Strategies for Sustaining Drug Release from Electrospun Nanofibers. J. Control. Release.

[39] Licciardello M., Ciardelli G., Tonda-Turo C. (2021). Biocompatible Electrospun Polycaprolactone-Polyaniline Scaffold Treated with Atmospheric Plasma to Improve Hydrophilicity. Bioengineering.

[40] Houchin M.L., Topp E.M. (2009). Physical Properties of PLGA Films during Polymer Degradation. J. Appl. Polym. Sci..

[41] Dias J.R., Sousa A., Augusto A., Bártolo P.J., Granja P.L. (2022). Electrospun Polycaprolactone (PCL) Degradation: An In Vitro and In Vivo Study. Polymers.

[42] Peng C., Zheng J., Chen D., Zhang X., Deng L., Chen Z., Wu L. (2018). Response of HPDLSCs on 3D Printed PCL/PLGA Composite Scaffolds in Vitro. Mol. Med. Rep..

[43] Critchley S., Sheehy E.J., Cunniffe G., Diaz-Payno P., Carroll S.F., Jeon O., Alsberg E., Brama P.A.J., Kelly D.J. (2020). 3D Printing of Fibre-Reinforced Cartilaginous Templates for the Regeneration of Osteochondral Defects. Acta Biomater..

[44] Zamanlui S., Mahmoudifard M., Soleimani M., Bakhshandeh B., Vasei M., Faghihi S. (2018). Enhanced Chondrogenic Differentiation of Human Bone Marrow Mesenchymal Stem Cells on PCL/PLGA Electrospun with Different Alignments and Compositions. Int. J. Polym. Mater. Polym. Biomater..

[45] Goonoo N., Bhaw-Luximon A., Jhurry D. (2014). Drug Loading and Release from Electrospun Biodegradable Nanofibers. J. Biomed. Nanotechnol..

[46] Caminal M., Peris D., Fonseca C., Barrachina J., Codina D., Rabanal R.M., Moll X., Morist A., García F., Cairó J.J. (2016). Cartilage Resurfacing Potential of PLGA Scaffolds Loaded with Autologous Cells from Cartilage, Fat, and Bone Marrow in an Ovine Model of Osteochondral Focal Defect. Cytotechnology.

[47] Qian Y., Chen H., Xu Y., Yang J., Zhou X., Zhang F., Gu N. (2016). The Preosteoblast Response of Electrospinning PLGA/PCL Nanofibers: Effects of Biomimetic Architecture and Collagen I. Int. J. Nanomed..

[48] Indolfi L., Baker A.B., Edelman E.R. (2012). The Role of Scaffold Microarchitecture in Engineering Endothelial Cell Immunomodulation. Biomaterials.

[49] Nicolas J., Magli S., Rabbachin L., Sampaolesi S., Nicotra F., Russo L. (2020). 3D Extracellular Matrix Mimics: Fundamental Concepts and Role of Materials Chemistry to Influence Stem Cell Fate. Biomacromolecules.

[50] Chiquet M., Gelman L., Lutz R., Maier S. (2009). From Mechanotransduction to Extracellular Matrix Gene Expression in Fibroblasts. Biochim. Biophys. Acta.

[51] Ijima H., Nakamura S., Bual R., Shirakigawa N., Tanoue S. (2018). Physical Properties of the Extracellular Matrix of Decellularized Porcine Liver. Gels.

[52] Brown B.N., Badylak S.F. (2014). Extracellular Matrix as an Inductive Scaffold for Functional Tissue Reconstruction. Transl. Res..

[53] Ishaug-Riley S.L., Okun L.E., Prado G., Applegate M.A., Ratcliffe A. (1999). Human Articular Chondrocyte Adhesion and Proliferation on Synthetic Biodegradable Polymer Films. Biomaterials.

[54] Jelodari S., Ebrahimi Sadrabadi A., Zarei F., Jahangir S., Azami M., Sheykhhasan M., Hosseini S. (2022). New Insights into Cartilage Tissue Engineering: Improvement of Tissue-Scaffold Integration to Enhance Cartilage Regeneration. BioMed Res. Int..

[55] Pawlik J., Łukowicz K., Cholewa-Kowalska K., Osyczka A.M. (2019). New Insights into the PLGA and PCL Blending: Physico-Mechanical Properties and Cell Response. Mater. Res. Express.

[56] Tanthaisong P., Imsoonthornruksa S., Ngernsoungnern A., Ngernsoungnern P., Ketudat-Cairns M., Parnpai R. (2017). Enhanced Chondrogenic Differentiation of Human Umbilical Cord Wharton’s Jelly Derived Mesenchymal Stem Cells by GSK-3 Inhibitors. PLoS ONE.

[57] Li L., Yu F., Zheng L., Wang R., Yan W., Wang Z., Xu J., Wu J., Shi D., Zhu L. (2019). Natural Hydrogels for Cartilage Regeneration: Modification, Preparation and Application. J. Orthop. Transl..

[58] Wei P., Xu Y., Zhang H., Wang L. (2021). Continued Sustained Insulin-Releasing PLGA Nanoparticles Modified 3D-Printed PCL Composite Scaffolds for Osteochondral Repair. Chem. Eng. J..

